# The influence of the intestinal microflora to the efficacy of Rosuvastatin

**DOI:** 10.1186/s12944-018-0801-x

**Published:** 2018-06-30

**Authors:** Lijun Wang, Yang Wang, Hongwei Wang, Xue Zhou, Xianjing Wei, Zezhou Xie, Zhipeng Zhang, Keke Wang, Jianjun Mu

**Affiliations:** 1 Department of Cardiology, First Affiliated Hospital of Xi’an Jiaotong University, Xi’an, 710061 China; 2 Department of Cardiology, Affiliated Zhongshan Hospital, Dalian University, Dalian, 116001 China; 3 Department of Laboratory, The Second Affiliated Hospital of Jiaxing University, Jiaxing Second Hospital, Jiaxing, 314000 China

**Keywords:** Intestinal flora, Rosuvastatin, Low density lipoprotein cholesterol, Probiotics

## Abstract

**Background:**

Intestinal microflora has been shown to play essential roles in the clinical therapies of metabolic diseases. The present study is aiming to investigate the potential roles and mechanisms of how intestinal microflora mediates lipid-reduction efficacy of Rosuvastatin.

**Methods:**

To investigate the correlation between the intestinal microflora and efficacy of Rosuvastatin, we analyzed the diversity of intestinal microflora using PCR-DGGE analysis and 16S rDNA sequencing approaches. Furthermore, we compared the blood lipid levels of rat models with dysbiosis of intestinal microflora and control rats upon the Rosuvastatin administration.

**Results:**

The diversity of the intestinal flora was obviously decreased upon the antibiotic treatment, this effect could be maintained for 2 weeks after establishment of the models. Importantly, the results from 16S rDNA sequencing demonstrated that the abundance of Lactobacillus and Bifidobacterium was remarkably diminished upon the antibiotic treatment in antibiotic+Rosuvastatin-treated group compared to that of Rosuvastatin-treated group and control group. Correspondently, the lipid-reduction efficacy of Rosuvastatin was significantly compromised. However, the diversity of the intestinal flora was recovered 4 weeks after the antibiotic treatment. Subsequently, the lipid-reduction efficacy of Rosuvastatin was also recovered to level of the control rats treated with Rosuvastatin alone.

**Conclusion:**

Intestinal flora could play an essential role in mediating the lipid-reduction efficacy of Rosuvastatin.

## Background

Coronary heart disease is the main threat to human health [1]. Cardiovascular events caused by coronary heart disease have become a worldwide concern. Statins, also known as HMG-CoA reductase inhibitors, are a class of lipid-reducing medications that are effective for both primary and secondary prevention for cardiovascular disease [2, 3]. Hydroxymethyl glutaric acid reductase (HMGCR) is the rate-limiting enzyme in the cholesterol synthesis pathway and plays an important role in the synthesis of cholesterol [4]. By inhibiting HMGCR, Statins reduce the synthesis of cholesterol in hepatocytes, up-regulate the low-density lipoprotein (LDL) receptor on hepatocytes’ membrane, accelerate LDL catabolism, thus decrease the blood levels of total cholesterol (TC) and low-density lipoprotein cholesterol (LDLc) [5]. Albeit effective application of Statins can significantly reduce the occurrence of cardiovascular events [2], many clinical investigations revealed that the efficacy of Statins on blood lipid levels are remarkably variable between individual patients.

It has been reported that many factors, such as genetic polymorphisms, exercise, smoking, diet, as well as nutraceuticals normally included within diet can significantly affected the blood lipid level and the efficacy of Statins on dyslipidemia [6]. However, the underlying mechanisms why different patients respond to the application of Statins differently are still unclear. Recently, accumulating studies revealed that intestinal microflora plays essential roles in metabolic diseases [7]. Impairment of intestinal microflora can exacerbate the body lipid metabolism disorders, as the flora-secreted bile acids, cholesterol, and mevalonate can inhibit the expression of HMGCR. Mechanistically, bacterial-derived bile acids correlate with the statin-induced LDLc reduction as well as the downregulation of HMGCR [8]. Furthermore, lactobacilli-produced hydroxymethyl glutaric acid, lactic acid, and uric acid can directly suppress the HMGCR expression [9]. Therefore, we hypothesis that abnormal intestinal flora might be another important factor affect the efficacy of Statins on different patients.

In this study, we established the rat model of dysbiosis of intestinal microflora by administration of antibiotics. By investigating the correlation between the diversity of the intestinal microflora and the blood lipid level upon Rosuvastatin treatment, we demonstrated the potential roles of the intestinal flora in regulating the efficacy of Statins, which can provide new insights in understanding of Statins’ efficacy on cardiovascular diseases.

## Methods

### Animals

In total thirty healthy male SPF SD rats (No. 211003700), weight (200 ± 20) g, were purchased from Center of Laboratory Animals, Dalian Medical University (animal certificate number: SYXK (Liao) 2013–0006). All the animal experiments were approved by the Animal ethics committee of Xi’an Jiaotong University.

### Establishment of dysbiosis of intestinal microflora model in rats

Ten male SPF SD rats were randomly selected for via gavage (*i.g.*) administration with Ceftriaxone sodium (2 g/kg body weight in 2 ml saline solution, twice/day; Roche China, 10,983,036) for 8 days according to a previously reported rat model for dysbiosis of intestinal microflora [10] and different animal dose conversion rate [11]. This group was classified as the Antibiotic+Statin group. The rest rats were randomly grouped into Statin group (*n* = 10) and Water group (*n* = 10), were *i.g.* administrated with 2 ml saline solution (twice/day) for 8 days.

### Administration of Rosuvastatin

The rats of Antibiotic+Statin group were *i.g.* administrated with Rosuvastatin (10 mg/kg body weight/day; AstraZeneca China, J20120006) dissolved in water for 4 weeks after 8 days administration of Ceftriaxone sodium. The rats from Statin group were *i.g.* administrated with Rosuvastatin (10 mg/kg body weight/day; AstraZeneca China, J20120006) for 4 weeks. The rats of control Water group were *i.g.* given same volume of water for 4 weeks.

### PCR-DGGE analysis

To evaluate the diversity of the intestinal microflora, the stool specimens were collected at 0, 2, and 4 weeks after Rosuvastatin administration and then were analyzed by PCR-Denaturing Gradient Gel Electrophoresis (DGGE) approaches. Briefly, the DNA templates were extracted from stool specimens using Stool DNA kit (Omega Bio-Tek, USA; D4015) according to the manufacturer’s protocol. The V3 region of 16S rRNAs was amplified using following primers: 5’-CCTACGGGAGGCAGCAG-3′; 5′- ATTACCGCGGCTGCTGG-3′. For DGGE analysis, the PCR products were run on 8% polyacrylamide gel and stained with GelRed for 30 min according to the manufacturer’s instruction (Bio-Rad Dcode System, USA). Then the gel was imaged using ChemiDocTM XRS+ system (Bio-Rad, USA) and analyzed using Quantity One software (Bio-Rad, USA).

### 16S rDNA-sequencing analysis

To assess the effects of Rosuvastatin on the intestinal microflora, the stool specimens were collected 2 weeks after administration of Rosuvastatin or water. The diversity of intestinal microflora was analyzed by sequencing the V3-V4 region of 16S rDNAs. The DNA samples were isolated as previously described. The DNA library was generated by two-step PCR approaches. First, the purified DNA templates were amplified using the following primers of 16S rDNA V3-V4 regions: 5′- ACTCCTACGGRAGGCAGCAG-3′ and 5′- GGACTACHVGGGTWTCTAAT-3′; secondly, the PCR products from first PCR step were purified using AxyPrep DNA Gel Extraction Kit (Axygen Scientific, USA; AP-GX-50) according to the manufacturer’s description, and then were performed for secondary PCR amplification to add the Illumina sequencing adaptors. The DNA libraries were purified further using AxyPrep DNA Gel Extraction Kit, and sequenced by TinyGene Bio-Tech(ShangHai)Co., Ltd. using Illumina MiSeq system (Illumina, USA). The Shannon analysis of the Alpha diversity of the intestinal microflora and the principle component analysis (PCA) were performed using mothur software (Version 1.33.2; https://www.mothur.org).

### Measurement of the blood lipid-related parameters

The blood samples were collected from the canthus of the eyes at the 0, 2 and 4 weeks after the administration of Rosuvastatin. The rats were starved for 12 h before the blood collection. The serum then was isolated, and the level of Triglyceride (TG), TC, high-density lipoprotein cholesterol (HDLc), and LDLc were determined via ADVIA 2400 Chemistry System (Siemens, Germany).

### Statistics

Statistical analysis was performed with SPSS 18.0. The data were presented as mean ± sem if not specifically described. The comparisons among more than 2 groups were performed using one-way ANOVA with Tukey’s multiple comparisons test. The comparisons between two groups were performed using unpaired two-tailed Student’s *t*-test. The statistically significant threshold was accepted as *p* < 0.05.

## Results

### The PCR-DGGE analysis of the animal model for dysbiosis of intestinal microflora

**Fig. 1:**
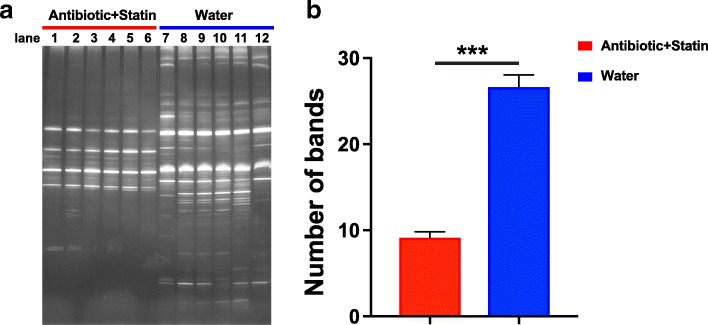
Ceftriaxone treatment disrupts diversity of the rat intestinal microflora. The photograph (**a**) and quantification (**b**) of the diversity of the intestinal microflora of rats from Ceftriaxone-treated Group Antibiotic+Statin (lane 1–6) and Control Group treated with water (lane 7–12) using PCR-DGGE analysis. The comparisons were performed using unpaired two-tailed Student’s *t*-test. The data were presented as mean ± sem. ***, *p* < 0.001

To investigate whether the intestinal microflora play roles in the efficacy of Rosuvastatin, we first established the rat model for dysbiosis of intestinal microflora by administrated heathy rats with antibiotic Ceftriaxone. To validate the dysbiosis of intestinal microflora model, we performed the PCR-DGGE analysis on the DNA samples extract from fresh stool specimens of rats treated with antibiotic and control rats without such treatment. Images and the quantification results suggested that Ceftriaxone treatment significantly decreased the diversity of intestinal microflora in Antibiotic+Statin group compared to water treated control group (Fig. 1a, b), indicating that antibiotic treatment remarkably impaired the diversity of the intestinal microflora in rats, mimicking the dysbiosis of intestinal microflora.

### Dysbiosis of intestinal microflora affects the efficacy of Rosuvastatin

**Fig. 2:**
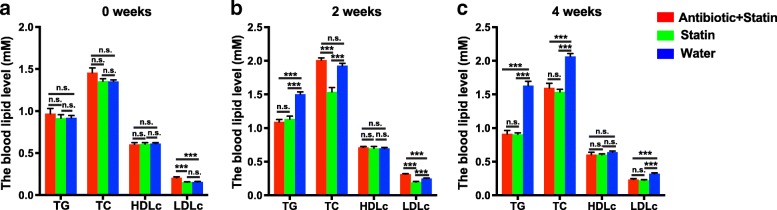
The blood lipid level at different time points of Rosuvastatin administration. **a**, the blood level of TG, TC, HDLc, and LDLc from different groups before Rosuvastatin treatment. **b**, the blood level of TG, TC, HDLc, and LDLc from different groups 2 weeks after Rosuvastatin treatment. **c**, the blood level of TG, TC, HDLc, and LDLc from different groups 4 weeks after Rosuvastatin treatment. The comparisons were performed using one-way ANOVA with Tukey’s multiple comparisons test. The data were presented as mean ± sem. ***, *p* < 0.001

To investigate whether the disruption of the intestinal microflora can influence the efficacy of Rosuvastatin on rats, we measured the blood level of TG, TC, HDLc, and LDLc at the 0, 2, and 4 weeks after administration of Rosuvastatin. Before the administration of Rosuvastatin (0 weeks), there was no difference in TG, TC, HDLc among different groups, while the treatment of antibiotic slightly increased the LDLc level compared to the LDLc level in rats from Statin Group and Water Group (Fig. 2a).

Two weeks after Rosuvastatin treatment, the blood levels of TG, TC, and LDLc from Statin Group were significantly reduced compared to control Water Group, implying the lipid-reduction effects of Rosuvastatin (Fig. 2b). However, the blood level of LDLc from Antibiotic+Statin Group are remarkable higher than the level from Statin Group and Water Group, and the blood level of TC are not down-regulated compared to control Water Group (Fig. 2b), suggesting that dysbiosis of intestinal microflora strongly affects the efficacy of Rosuvastatin on lipid-reduction. Compared to that from Water Group, blood level of LDLc from Statin Group was significantly decreased, suggesting that LDLc was reduced in conventional Statin Group. In addition, the blood level of TG showed no difference between Antibiotic+Statin Group and Statin Group ((Fig. 2b), indicating that alternation of intestinal microflora did not influent the effect of Rosuvastatin on TG-reduction.

Interestingly, 4 weeks after Rosuvastatin administration, the blood levels of TG, TC and LDLc from both Antibiotic+Statin Group and Statin Group were significantly lower than the levels from control Water Group (Fig. 2c). There was no statistical difference observed between Antibiotic+Statin Group and Statin Group (Fig. 2c). These results suggested that the influence of alternation of intestinal microflora on the efficacy of Rosuvastatin recovered alone the treatment. However, whether the recovery of the efficacy of Rosuvastatin effect on lipid-reduction are due to the rehabilitation of intestinal microflora was still unclear.

### Efficacy of Rosuvastatin on lipid-reduction correlated with the diversity of intestinal microflora

**Fig. 3:**
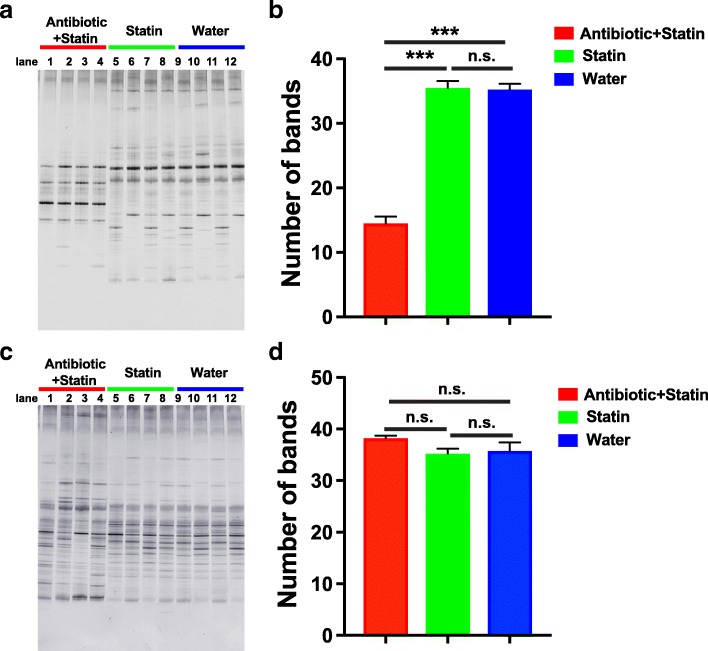
The diversity of intestinal microflora analysis at different time points after Rosuvastatin administration. **a** and **b**, the photograph (**a**) and quantification (**b**) of the diversity of the intestinal microflora of rats from Ceftriaxone-treated Group Antibiotic+Statin (lane 1–4), Group Statin (lane 5–8) and Control Group Water (lane 9–12) using PCR-DGGE analysis 2 weeks after Rosuvastatin treatment. C and D, the photograph (**c**) and quantification (**d**) of the diversity of the intestinal microflora of rats from Ceftriaxone-treated Group Antibiotic+Statin (lane 1–4), Group Statin (lane 5–8) and Control Group Water (lane 9–12) using PCR-DGGE analysis 4 weeks after Rosuvastatin treatment. The comparisons were performed using one-way ANOVA with Tukey’s multiple comparisons test. The data were presented as mean ± sem. ***, *p* < 0.001

To investigate whether the efficacy of Rosuvastatin is correlated with the intestinal microflora, the diversity of intestinal microflora was analyzed at 2 weeks and 4 weeks after Rosuvastatin treatment using PCR-DGGE approaches. Results from 2 weeks after Rosuvastatin treatment revealed that the diversity of intestinal microflora from Antibiotic+Statin Group are highly reduced compared to that from Statin Group and Water Group (Fig. 3a, b). However, the diversity of intestinal microflora from Antibiotic+Statin Group are fully restored to the normal level as Statin Group and Water Group 4 weeks after treatment (Fig. 3c, d). Taken together with the blood lipid levels from 2 and 4 weeks after Rosuvastatin treatment, those observation demonstrated that the intestinal microflora play essential roles in the efficacy of Rosuvastatin on lipid-reduction.

### Lactobacillu and Bifidobacterium might play roles in efficacy of Rosuvastatin treatment

**Fig. 4:**
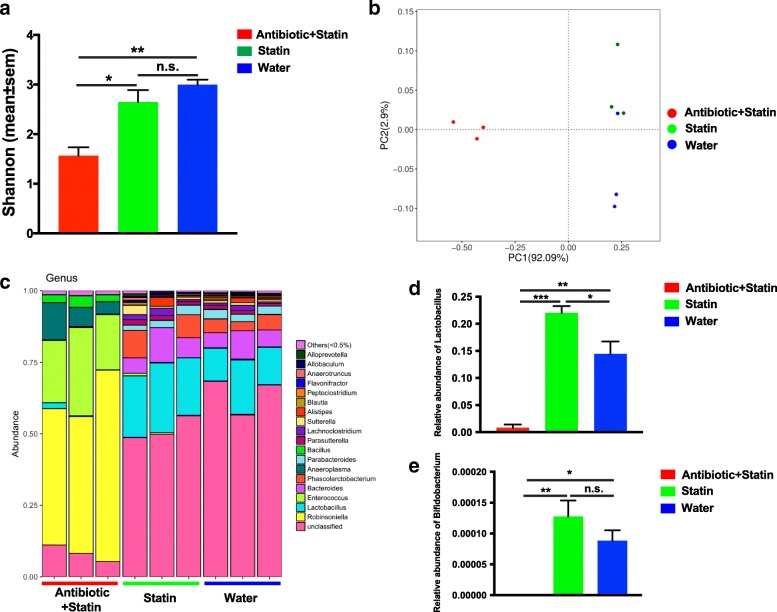
The composition of major intestinal microflora 2 weeks after Rosuvastatin treatment. **a**, Shannon analysis showed that the diversity of intestinal microflora from Group Antibiotic+Statin are significantly reduced compared to Group Statin and Water. **b**, Principle component analysis of the major composition of the intestinal microflora from different groups. The major population of intestinal microflora are remarkably changed upon the antibiotic treatment. **c**, Bray analysis of the major intestinal microflora from different groups. **d**, Lactobacillu is remarkably diminished upon the antibiotic treatment. **e**, Bifidobacterium is remarkably reduced upon the antibiotic treatment. The comparisons were performed using one-way ANOVA with Tukey’s multiple comparisons test. The data were presented as mean ± sem. *, *p* < 0.01; **, *p* < 0.05; ***, *p* < 0.001

To further investigate the potential floras which might play important roles in the lipid-reduction efficacy of Rosuvastatin, we analyzed the major composition of intestinal microflora from different groups 2 weeks after Statin treatment. Via 16S rDNA sequencing, we analyzed the major floras, including robinsoniella, lactobacillus, enterococcus, phascolarctobacterium, anaeroplasma, bacteroides, parabacteroides, bacillus, sutterella, lachnoclostridium, alistipes, parasutterella, flavonifactor, allobaculum, peptoclostridium, allopreotella, anaerotruncus, and bifidobacterium, et al. (Table 1). The Shannon analysis of Alpha diversity of the major composition of intestinal microflora between different groups showed that the Shannon index of rats from Antibiotic+Statin Group were dramatically decreased compared to Statin Group and Water Group (Fig. 4a). Similarly, principle component analysis of the abundance of intestinal microflora also indicated that the major population of intestinal microflora from Antibiotic+Statin Group are extremely different from that of Statin Group and Water Group (Fig. 4b), while the major intestinal microflora from Statin Group and Water Group are similar (Fig. 4b). These observations demonstrated that the antibiotic-treatment dramatically changed the major population of intestinal microflora. Bray analysis of the composition of major floras further confirmed PCA analysis: the intestinal microflora from Antibiotic+Statin Group are highly distinct from Statin Group and Water Group, which share the similar flora compositions (Fig. 4c). In addition, among the major microflora, Lactobacillu and Bifidobacterium are remarkably diminished upon the antibiotic treatment compared to the abundance of those from Statin Group, implying their potential roles in lipid-reduction efficacy of Rosuvastatin (Table 1, Fig. 4d, e).

**Table 1:** The analysis of the diversity of rat intestinal microflora (mean ± sem)

	Antibiotic+Statin	Statin	Water
Robinsoniella	5.41E-01 ± 6.40E-02^∆∆∆/^***	2.24E-03 ± 1.13E-03^n.s.^	1.44E-03 ± 4.17E-04
Lactobacillus	8.26E-03 ± 5.96E-03^∆∆∆/^**	2.20E-01 ± 1.25E-02^#^	1.45E-01 ± 2.28E-02
Enterococcus	2.40E-01 ± 3.50E-02^∆∆∆/^***	4.12E-03 ± 2.59E-03^n.s.^	2.16E-03 ± 7.35E-04
Bacteroides	2.71E-03 ± 2.21E-04^∆/^*	8.18E-02 ± 2.04E-02^n.s.^	7.05E-02 ± 1.45E-02
Phascolarctobacterium	1.86E-04 ± 8.38E-05^n.s./ n.s.^	5.86E-02 ± 2.93E-02^n.s.^	4.47E-02 ± 6.63E-03
Anaeroplasma	7.97E-02 ± 2.58E-02^∆/^*	3.92E-05 ± 2.60E-05^n.s.^	4.91E-05 ± 9.81E-06
Parabacteroides	3.53E-04 ± 4.50E-05^∆∆/^**	2.54E-02 ± 4.63E-03^n.s.^	2.93E-02 ± 2.21E-03
Bacillus	3.01E-02 ± 5.06E-03^∆∆∆/^***	7.36E-04 ± 2.64E-04^n.s.^	5.50E-04 ± 1.04E-04
Parasutterella	3.93E-05 ± 1.96E-05^∆∆∆/^**	1.57E-02 ± 1.84E-03^n.s.^	1.22E-02 ± 1.65E-03
Lachnoclostridium	5.89E-05 ± 4.50E-05^n.s./ n.s.^	1.69E-02 ± 5.69E-03^n.s.^	1.00E-02 ± 3.68E-03
Sutterella	1.96E-05 ± 9.81E-06^n.s./ n.s.^	1.56E-02 ± 8.14E-03^n.s.^	8.58E-03 ± 7.17E-04
Alistipes	3.34E-04 ± 8.38E-05^n.s./ n.s.^	1.26E-02 ± 9.48E-03^n.s.^	9.21E-03 ± 3.73E-03
Blautia	9.81E-06 ± 9.81E-06^n.s./ n.s.^	4.36E-03 ± 2.55E-03^n.s.^	3.98E-03 ± 6.99E-04
Peptoclostridium	1.96E-05 ± 1.96E-05^∆∆/^**	3.96E-03 ± 1.03E-03^n.s.^	4.19E-03 ± 1.96E-04
Flavonifractor	9.81E-06 ± 9.81E-06^∆∆/^*	4.17E-03 ± 6.46E-04^n.s.^	2.81E-03 ± 6.32E-04
Anaerotruncus	7.85E-05 ± 3.54E-05^n.s./ n.s.^	3.55E-03 ± 3.12E-03^n.s.^	3.31E-03 ± 8.36E-04
Allobaculum	4.91E-05 ± 2.60E-05^n.s./ n.s.^	4.29E-03 ± 1.85E-03^n.s.^	2.57E-03 ± 9.12E-04
Bifidobacterium	0.00E + 00 ± 0.00E + 00^∆∆/^*	1.28E-04 ± 2.60E-05^n.s.^	8.83E-05 ± 1.70E-05

∆ Antibiotic+Statin v.s. Statin

*Antibiotic+Statin v.s. Water

#Statin v.s. Water

*, Δ, # *p* < 0.05 **, ΔΔ *p* < 0.01 ***, ΔΔΔ *p* < 0.001

## Discussion

The intestinal microflora is a vast and complex ecosystem that includes more than 1000 species [12]. Most physiological functions of human body are the result of symbiosis formed during coevolution of human body and normal microflora. The intestinal microflora plays important physiological roles in many biological processes, including nutrient absorption, organ growth and development, metabolism, and so on [13, 14]. However, the balance of intestinal flora can be disrupted easily by various external factors, such as diet, probiotics and antibiotics. Imbalance of intestinal microflora could cause severe functional disorders and diseases. Recent studies also indicated that the intestinal microflora was involved in the efficacy of medicines on different metabolic diseases [15].

It is well known that Statins are widely used in the prevention and treatment of atherosclerotic diseases mainly by inhibiting HMGCR and lowering LDLc [16, 17]. Their lipid-lowering efficacy is reported to be affected by many factors such as genetic variations and lifestyle habits [18, 19]. Kaddurah-Daouk et al. found a clear correlation between gut-derived metabolites and Simvastatin response [8]. Studies by Yoo et al. found that antibiotic intake might reduce the intestinal flora’s biotransformation of oral lovastatin, and subsequent effects on microbial metabolism might lead to systemic changes in the drug and their function [20]. So far, there is no reports described the correlation between intestinal flora and the lipid-reduction efficacy of Rosuvastatin. In this study, we investigated the roles of intestinal microflora in the blood lipid-reduction efficacy of Rosuvastatin.

Ceftriaxone sodium is a broad-spectrum third-generation cephalosporin antibiotic that has strong antibacterial activity against most Gram-positive and -negative bacteria. It is also recognized as one of those important antibiotics which induce the imbalance of intestinal microflora [21]. In this study, we confirmed that high dose ceftriaxone treatment dramatically reduced the diversity of intestinal flora disorder. In addition, the level of LDLc from antibiotic-treated group was significantly higher than the other groups without antibiotic treatment, suggesting the broad-spectrum antibiotic-induced decrease of intestinal microflora may increase the blood level of LDLc. Indeed, previous studies implied that ceftriaxone administration could induce the reduction of the intestinal Enterobacteriaceae, Lactobacillus, and Bifidobacterium [22, 23], which were negatively associated with the levels of TC, non-HDLc, and anti-oxidant LDL (oxLDL Ab) in the blood [24].

Two weeks after Rosuvastatin, PCR-DGGE analysis showed that compared to Statin Group and the control Water Group, the diversity of intestinal flora diversity of rats in Antibiotic+Statin group was still significantly reduced. Correspondingly, the blood level of LDLc and TC from Antibiotic+Statin Group are remarkable higher than the level from Statin Group, suggesting that dysbiosis of intestinal microflora strongly affects the efficacy of Rosuvastatin on lipid-reduction. The blood levels of TG, TC and LDLc from Statin Group were significantly reduced compared to control Water Group, implying the lipid-reduction effects of Rosuvastatin. Interestingly, 4 weeks after Statin administration, PCR-DGGE analysis showed no significant difference in the diversity of intestinal flora among all three groups, suggesting that the alternation of intestinal microflora caused by antibiotic treatment was reestablished. Accordingly, the level of LDLc and TC in Group Antibiotic+Statin showed no statistical difference compared with the level from Statin Group, but dramatically reduced compared to that from the control Water Group, indicating that the efficacy of Rosuvastatin on LDLc and TC was recovered along with the restoration of intestinal microflora. Further investigation of 16S rDNA revealed that average abundance of probiotic bacteria in Group Antibiotic+Statin are dramatically reduced compared to Statin and Water Group. Previous studies showed that Lactobacilli have the effect of lowering cholesterol [25–27]. Therefore, the serious reduction of intestinal lactobacilli in the antibiotic statin group, will affect the synthesis and metabolism of low cholesterol, leading to the abnormal increase of low density lipoprotein cholesterol in this group before treatment and 2 weeks after Statin treatment. The blood level of TG among three groups are not altered before Statin treatment, while it is reduced both in Antibiotic+Statin Group and Statin Group 2 and 4 weeks after treatment, indicating that intestinal flora does not affect Rosuvastatin’s effects on reducing TG. The blood level of HDLc showed no difference among three groups before and after Statin administration, indicating that Rosuvastatin showed no effect on HDLc.

Rosuvastatin is an inhibitor of HMGCR, which targeting liver cholesterol de novo synthesis [28]. But only about 10% of Rosuvastatin calcium is metabolized by liver CYP450, and most of Rosuvastatins are excreted in its original form [29]. Therefore, it is possible that the Rosuvastatin could exert impacts on the gut flora. Our study clearly showed that with the antibiotic treatment, Rosuvastatin lost its anti-cholesterol effects and regained it after antibiotic withdraw. Thus, to our knowledge, we might prove for the first time that gut microbiome is important for therapeutic effects of Rosuvastatin.

It is not novel that gut microbiome could interfere the host cholesterol metabolism. 16S rDNA sequencing results in our study showed that Rosuvastatin treatment for 2 weeks, significantly reduced Lactobacillus and Bifidobacterium population in Antibiotic+Statin group. Various studies have shown that the change of intestinal probiotics Bifidobacterium and Lactobacillus can lead to the alternation of TNFα and IL-6 levels, which result in the reduction of cholesterol [30]. In addition, the expression of one cellular transporter for Rosuvastatin, organic anion transporting polypeptide 1B1 (OATP1B1), is regulated by a variety of factors including TNFα and IL6 [31]. Thus, the potential mechanism that mediate microbiome impact on Rosuvastatin effects might due to the transportation defects caused by diminished population of intestinal Lactobacillus and Bifidobacterium.

## Conclusions

Taken together, our study showed that the disorder of intestinal flora can significantly reduce the efficacy of Rosuvastatin on lowing blood level of TC and LDLc in SD rats. After the recovery of intestinal flora, the Rosuvastatin effect on lipid-reduction returned to normal level. Thus, the effect of Rosuvastatin on lowering TC and LDLc can be affected by the intestinal flora. The potential mechanism is considered to associate with the increase of inflammatory factors induced by disorder of intestinal flora, and the higher expression of transporters, and the reduction of beneficial bacteria.

## Abbreviations

DGGE: Denaturing Gradient Gel Electrophoresis
HDLc: High-density lipoprotein cholesterol
HMGCR: Hydroxymethyl glutaric acid reductase
LDL: Low-density lipoprotein
LDLc: Low-density lipoprotein cholesterol
OATP1B1: Organic anion transporting polypeptide 1B1
PCA: Principle component analysis
TC: Total cholesterol
TG: Triglyceride

## References

[1] GBD 2015 Mortality and Causes of Death Collaborators (2016). Global, regional, and national life expectancy, all-cause mortality, and cause-specific mortality for 249 causes of death, 1980-2015: a systematic analysis for the global burden of disease study 2015. Lancet.

[2] Taylor F, Huffman MD, Macedo AF, Moore TH, Burke M, Davey Smith G (2013). Statins for the primary prevention of cardiovascular disease. Cochrane Database Syst Rev.

[3] National Clinical Guideline Centre (UK) (2014). Lipid modification: cardiovascular risk assessment and the modification of blood lipids for the primary and secondary prevention of cardiovascular disease.

[4] Brown MS, Goldstein JL (1980). Multivalent feedback regulation of HMG CoA reductase, a control mechanism coordinating isoprenoid synthesis and cell growth. J Lipid Res.

[5] Chou R, Dana T, Blazina I, Daeges M, Jeanne TL (2016). Statins for prevention of cardiovascular disease in adults: evidence report and systematic review for the US preventive services task force. JAMA.

[6] Scicchitano P, Cameli M, Maiello M, Modesti PA, Muiesan ML, Novo S (2014). Nutraceuticals and dyslipidaemia: beyond the common therapeutics. J functional. Foods.

[7] Daliri EB, Wei S, Oh DH, Lee BH (2017). The human microbiome and metabolomics: current concepts and applications. Crit Rev Food Sci Nutr.

[8] Kaddurah-Daouk R, Baillie RA, Zhu H, Zeng ZB, Wiest MM, Nguyen UT (2011). Enteric microbiome metabolites correlate with response to simvastatin treatment. PLoS One.

[9] Parks DJ, Blanchard SG, Bledsoe RK, Chandra G, Consler TG, Kliewer SA (1999). Bile acids:natural ligands for an orphan nuclear receptor. Science.

[10] Luo X, Zheng Y, Wen R, Deng X, Zhou L, Liao H (2016). Effects of ceftriaxone induced intestinal dysbacteriosis on lymphocytes in different tissues in mice. Immunobiology.

[11] Nair AB, Jacob S (2016). A simple practice guide for dose conversion between animals and human. J Basic Clin Pharm.

[12] Sears CL (2005). A dynamic partnership: celebrating our gut flora. Anaerobe.

[13] Gill SR, Pop M, Deboy RT, Eckburg PB, Turnbaugh PJ, Samuel BS (2006). Metagenomic analysis of the human distal gut microbiome. Science.

[14] Gonzalez FJ, Nebert DW (1990). Evolution of the P450 gene superfamily: animal-plant ‘warfare’, molecular drive and human genetic differences in drug oxidation. Trends Genet.

[15] Thomas S, Izard J, Walsh E, Batich K, Chongsathidkiet P, Clarke G (2017). The host microbiome regulates and maintains human health: a primer and perspective for non-microbiologists. Cancer Res.

[16] Baigent C, Keech A, Kearney PM, Blackwell L, Buck G, Pollicino C (2005). Efficacy and safety of cholesterol-lowering treatment: prospective meta-analysis of data from 90,056 participants in 14 randomised trials of statins. Lancet.

[17] de Vries FM, Kolthof J, Postma MJ, Denig P, Hak E (2014). Efficacy of standard and intensive statin treatment for the secondary prevention of cardiovascular and cerebrovascular events in diabetes patients: a meta-analysis. PLoS One.

[18] Barber MJ, Mangravite LM, Hyde CL, Chasman DI, Smith JD, McCarty CA (2010). Genome-wide association of lipid-lowering response to statins in combined study populations. PLoS One.

[19] Thompson GR, O'Neill F, Seed M (2002). Why some patients respond poorly to statins and how this might be remedied. Eur Heart J.

[20] Yoo DH, Kim IS, Van Le TK, Jung IH, Yoo HH, Kim DH (2014). Gut microbiota-mediated drug interactions between lovastatin and antibiotics. Drug Metab Dispos.

[21] Damrongmanee A, Ukarapol N (2007). Incidence of antibiotic-associated diarrhea in a pediatric ambulatory care setting. J Med Assoc Thail.

[22] Chen DC, Ma LQ, Liu SZ (2009). Effects of rhubarb on intestinal flora and bacterial translocation in rats with sepsis. Zhongguo Wei Zhong Bing Ji Jiu Yi Xue.

[23] Welling GW, Meijer-Severs GJ, Helmus G, van Santen E, Tonk RH, de Vries-Hospers HG (1991). The effect of ceftriaxone on the anaerobic bacter flora and the bacterial enzymatic activity in the intestinal tract. Infection.

[24] Cavallini DC, Suzuki JY, Abdalla DS, Vendramini RC, Pauly-Silveira ND, Roselino MN (2011). Influence of a probiotic soy product on fecal microbiota and its association with cardiovascular risk factors in an animal model. Lipids Health Dis.

[25] Liu Y, Zhao F, Liu J, Wang H, Han X, Zhang Y (2017). Selection of cholesterol-lowering lactic acid Bacteria and its effects on rats fed with high-cholesterol diet. Curr Microbiol.

[26] Singh TP, Malik RK, Katkamwar SG, Kaur G (2015). Hypocholesterolemic effects of lactobacillus reuteri LR6 in rats fed on high-cholesterol diet. Int J Food Sci Nutr.

[27] Xie N, Cui Y, Yin YN, Zhao X, Yang JW, Wang ZG (2011). Effects of two lactobacillus strains on lipid metabolism and intestinal microflora in rats fed a high-cholesterol diet. BMC Complement Altern Med.

[28] Kunze A, Huwyler J, Camenisch G, Poller B (2014). Prediction of organic anion-transporting polypeptide 1B1- and 1B3-mediated hepatic uptake of statins based on transporter protein expression and activity data. Drug Metab Dispos.

[29] Schuster H (2003). Rosuvastatin--a highly effective new 3- hydroxy-3-methylglutaryl coenzyme A reductase inhibitor: review of clinical trial data at 10–40 mg doses in dyslipidemic patients. Cardiology.

[30] Veiga P, Juste C, Lepercq P, Saunier K, Béguet F, Gérard P (2005). Correlation between faecal microbial community structure and cholesterol-to-coprostanol conversion in the human gut. FEMS Microbiol Lett.

[31] Le Vee M, Lecureur V, Stieger B, Fardel O (2009). Regulation of drug transporter expression in human hepatocytes exposed to the proinflammatory cytokines tumor necrosis factor-alpha or interleukin-6. Drug Metab Dispos.

